# Intermuscular Fat: A Review of the Consequences and Causes

**DOI:** 10.1155/2014/309570

**Published:** 2014-01-08

**Authors:** Odessa Addison, Robin L. Marcus, Paul C. LaStayo, Alice S. Ryan

**Affiliations:** ^1^Division of Gerontology and Geriatric Medicine, Department of Medicine, University of Maryland School of Medicine, 10 North Green Street, BT/18/GRECC, Baltimore, MD 21201, USA; ^2^Geriatric Research, Education and Clinical Center, Baltimore Veterans Affairs Medical Center, Baltimore, MD 21201, USA; ^3^Department of Physical Therapy, University of Utah, Salt Lake City, UT 84108, USA; ^4^Department of Exercise and Sport Science, University of Utah, Salt Lake City, UT 84112, USA; ^5^Department of Orthopedics, University of Utah, Salt Lake City, UT 84108, USA

## Abstract

Muscle's structural composition is an important factor underlying muscle strength and physical function in older adults. There is an increasing amount of research to support the clear disassociation between the loss of muscle lean tissue mass and strength with aging. This disassociation implies that factors in addition to lean muscle mass are responsible for the decreases in strength and function seen with aging. Intermuscular adipose tissue (IMAT) is a significant predictor of both muscle function and mobility function in older adults and across a wide variety of comorbid conditions such as stroke, spinal cord injury, diabetes, and COPD. IMAT is also implicated in metabolic dysfunction such as insulin resistance. The purpose of this narrative review is to provide a review of the implications of increased IMAT levels in metabolic, muscle, and mobility function. Potential treatment options to mitigate increasing levels of IMAT will also be discussed.

## 1. Introduction

The unique ability of adipose tissue to expand throughout life and release a host of chemical messengers makes adipose not only a distinctive tissue but also the largest endocrine organ in the body [1]. In the last twenty years, a rapid expansion of our understanding of this unique organ has occurred. Once thought to be an inert storage depot for excess calories, important only to energy homeostasis, we now know that adipose tissue expresses and secretes a multitude of hormones and proinflammatory cytokines thereby acting in an autocrine, paracrine, and endocrine manner signaling the heart, musculoskeletal, central nervous, and metabolic systems [1–3]. Not all adipose depots are alike. Recent studies have suggested that the location [4–8] and type [9] of excess adipose tissue, rather than simply total body adiposity, may be important in the systemic increase of circulating cytokines and the rise in metabolic diseases such as diabetes [9–14] (for a more complete review of the types and roles of adipose tissue, see Wronska 2012 and Stehno-Bittel 2008) [1, 9]. Adipose tissue stored in subcutaneous depots, particularly in the gluteal-femoral region, is a negative predictor of metabolic syndrome and is cardioprotective [4–7, 15, 16]. However, adipose tissue stored in ectopic locations outside of the subcutaneous tissue such as in the muscle, liver, and abdominal cavity is linked with chronic inflammation [10, 17–19], impaired glucose tolerance [4–6, 20, 21], increased total cholesterol [8, 16, 22], and decreased strength and mobility in older adults [23–31]. Advancing age results in a redistribution of fat depots, despite stable or decreasing overall fat, with adipose storage sites changing from subcutaneous locations to the more harmful ectopic locations [3, 32, 33]. In particular, intermuscular adipose tissue (IMAT), an ectopic fat depot found beneath the fascia and within the muscles, may be of specific interest to rehabilitation professionals.

IMAT has been studied in a variety of individuals with metabolic [5, 6, 8, 14, 28, 34–36], orthopedic [37, 38], and neurologic [39, 40] conditions commonly seen in rehabilitative settings. High levels of IMAT are associated with insulin resistance [5, 6, 8, 14, 28, 34–36], a loss of strength [23–31], and mobility dysfunction [23, 41–43]. High levels of IMAT can be found in many patient populations, including, but not restricted to, the paraspinal muscles of individuals with chronic back pain [37, 38] and the locomotor muscles of individuals diagnosed with HIV [44], spinal cord injury [39], CVA [40], diabetes [6], and COPD [45]. Furthermore, older adults with increased IMAT levels in the locomotor muscles are known to experience increased levels of muscle weakness, decreased mobility function [23, 41–43], and an increased risk of future mobility limitation [42, 43]. IMAT has potential clinical implications that rehabilitation professionals should recognize and attempt to manage in rehabilitation settings when working with older adults and those with diseases and disabilities associated with IMAT.

The purpose of this narrative review is to inform rehabilitation professionals about the potential metabolic, muscle, and mobility associations of increased IMAT in the locomotor muscles of adults. This review will focus on three areas. First, the definition and measurement of IMAT will be presented; second, the implications of increased locomotor muscle IMAT in metabolism, muscle strength, and mobility will be reviewed; and third, recommendations for future research and treatment for adults with increased levels of IMAT will be made. Literature targeted for this review included peer reviewed cross-sectional, longitudinal, epidemiologic, and clinical studies in adult humans.

## 2. Definitions and Measurements of IMAT

**Figure 1:**
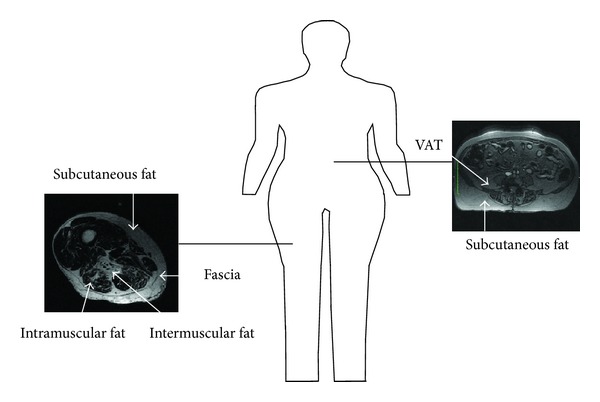
Intermuscular fat is generally considered to be any fat (including the fat between muscle groups and within a muscle) found beneath the fascia of a muscle and is the widest definition for fat beneath the fascia of a muscle. Intramuscular fat is the visible fat found within a muscle. Intermuscular is considered to be an ectopic fat depot similar to visceral adipose tissue (VAT) found in the abdomen.

IMAT has been referred to in the literature by a variety of names and definitions including myostasis, intermuscular fat, intramuscular fat, and low density lean tissue. Intermuscular fat is typically the broadest definition of fatty infiltration in the muscle referring to storage of lipids in adipocytes underneath the deep fascia of muscle. This includes the visible storage of lipids in adipocytes located between the muscle fibers (also termed intramuscular fat) and also between muscle groups (literally intermuscular) [46] (See Figure 1). While not frequently isolated as a separate fat depot by itself, there also exists a smaller group of lipids stored within the muscle cells themselves known as intramyocellular lipids or IMCL; IMCL has been reviewed extensively elsewhere [47]. Increased levels of IMCL are found both in obese insulin resistant individuals and in highly trained endurance athletes; these paradoxical findings have led to the conclusion that lipids stored within muscle cells are not always harmful to the cell [47]. For the remainder of this review, the term IMAT will refer to any measure of fat beneath the deep fascia of the thigh, not including studies that have used methods that independently quantify IMCLs (i.e., histochemical or spectroscopic methods).

IMAT is most commonly measured via computed tomography (CT) or magnetic resonance imaging (MRI). While IMAT has been quantified in numerous studies, it is not yet routinely measured or quantified in clinical imaging studies. CT scans have been extensively used to quantify IMAT in numerous studies [5, 6, 10, 14, 20, 23, 28, 40, 42, 43, 48–52] and were first described by Kelley et al. in 1991 [53]. CT is a fast imaging method that utilizes X-rays for an indirect measurement of IMAT based on the tissue density of an area. On a continuum of density where bone is the most dense and fat is the least dense, lean muscle mass falls between these two extremes. Lean tissue seen on a CT scan can be further divided into areas of high-density lean tissue and areas of low-density lean tissue. High-density lean is an area where little fatty infiltration occurs, and low-density lean tissue is an area where increased levels of adipocytes are found between and within muscle fibers and result in decreased density on CT scan. An individual with a higher proportion of low-density lean is assumed to have increased levels of both IMCL and IMAT. If the density of a muscle increases, or the area of low-density lean decreases after an exercise program, it is presumed that the exercise program has resulted in a loss of both IMCL and IMAT.

With MRI, direct measurements of IMAT [46] can occur without the use of harmful radiation; therefore, MRI is increasingly used to quantify IMAT [25–27, 29–31, 35, 36, 39, 46, 54–65]. MRIs utilize the chemical properties of fat and muscle to directly measure the amount of IMAT within a region of interest [46]. However, while MRI studies of IMAT avoid the use of harmful radiation, they do typically require time-consuming manual segmentation for a region of interest. This process can be difficult and less reliable for small, irregularly shaped areas. Comparative studies of MRI and CT have demonstrated that MRI has a higher sensitivity than CT for identifying early fatty replacement in muscle and that MRI, because it is not density based, provides better anatomical details of soft tissue than CT [46, 66, 67]. Studies comparing CT and MRI measurements have generally shown good agreement and both methods are acceptable precise measures of IMAT [68, 69]. The same definition and method for measuring IMAT should be used in pre- and poststudies. Both CT and MRI appear to be appropriate and advanced techniques for measuring IMAT; however, drawing conclusions concerning absolute amounts of IMAT across studies may be difficult if different methods of measurement are employed. Many studies have used slightly different definitions of IMAT (i.e., adipose tissue in a muscle, adipose tissue between muscles, or adipose tissue under the fascia of the thigh), and conclusions drawn across studies should be interpreted within this context.

## 3. IMAT and Metabolism

**Figure 2:**
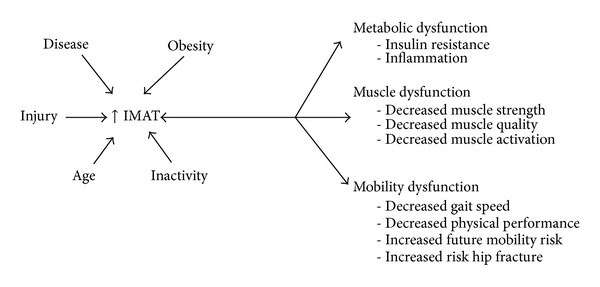
Muscle injury, obesity, age, disease status, and inactivity are all factors that are associated with increased levels of IMAT. Increased levels of IMAT may also lead to a myriad of metabolic, muscle, and mobility dysfunctions.

IMAT is positively associated with insulin resistance and an increased risk of developing type 2 diabetes [5, 6, 8, 14, 28, 34–36] (Figure 2). The link between IMAT and insulin resistance could be theoretically attributed to the relationship of IMAT and BMI. Generally, as BMI increases so does IMAT [7, 21, 23]. However, even when BMI is statistically accounted for, IMAT remains a strong predictor of fasting glucose and insulin levels in both younger [5] and older adults [6, 22, 54], suggesting that these metabolic impairments are not simply due to obesity alone. Compared to subcutaneous fat, IMAT is a much smaller fat depot, accounting for as little as 8% of the adipose tissue in the thigh [5]. Despite its small size, IMAT is strongly associated with insulin sensitivity in obese individuals [5]. It is currently unknown if IMAT acts merely as a marker of metabolic dysfunction or if it may have an intermediary or modifying role in insulin resistance. Since IMAT sits in close proximity to the muscle fibers, it is possible that IMAT may interact with muscle fibers through a yet unknown pathway leading to muscle dysfunction and insulin resistance [10, 26]. Muscle dysfunction may lead to further inactivity and increased levels of IMAT precipitating a cycle of increased IMAT, insulin resistance, and muscle dysfunction. This close relationship between the muscle fibers and IMAT becomes particularly important in populations that are known to have increased IMAT, muscle dysfunction, and insulin resistance including individuals with diabetes [70] and survivors of stroke [28, 40] and spinal cord injury [39, 57, 58].

After a stroke (CVA), muscle volume decreases and both subcutaneous adipose tissue and IMAT increase in the paretic limb [28, 40]. We noted that, in the paretic limb, the subcutaneous adipose depot was 6% higher and IMAT was increased 4% compared to the nonparetic limb in older stroke survivors [28]. Similar to the findings in older adults with type 2 diabetes, a positive relationship also exists between IMAT and fasting insulin levels in those post-CVA [28]. In this study of 70 adult stroke survivors, we found that decreased muscle attenuation (indicating increased IMAT levels) was associated with increased fasting insulin levels [28]. Similar results are found in those who have suffered a spinal cord injury. One study found that thigh IMAT increased on average 26% in just three months after a complete spinal cord injury [39]. This large increase in IMAT accounted for a 70% reduction in glucose tolerance in these same individuals [39]. The strong relationship observed between decreased glucose tolerance and increased IMAT postspinal cord injury suggests that accumulation of IMAT may have a deleterious effect on glucose homeostasis particularly in those who are mobility limited. Further studies are necessary to determine if IMAT plays a direct role in decreased glucose tolerance or if it is only a marker of metabolic dysfunction.

Despite not knowing the specific mechanism behind IMAT's potentially harmful influence on muscle metabolism, there are several lines of evidence that support this relationship. Multiple authors have suggested that IMAT, an ectopic fat depot similar to visceral adipose tissue, may release a host of proinflammatory cytokines resulting in local inflammation within the muscle [10, 26, 48, 65, 71]. Other ectopic fat depots, such as those found in the liver or the abdomen, are known to have increased systemic levels of proinflammatory cytokines [72]. Beasley et al. also reported a relationship between the amount of IMAT within the thigh and systemic measures of proinflammatory cytokines, as measured in the serum suggesting that IMAT may in fact be related to increased whole body inflammation [10]. We reported for the 1st time increased IMAT in the paretic leg of stroke survivors [40], which we followed with our examination of skeletal muscle TNF-a [73]. We found that both IMAT [40] and inflammation [73] are increased in the paretic leg of stroke survivors [28, 73]. However, to date, we are unaware of any published examinations of the direct relationship between IMAT and the local inflammatory environment within the muscle. Skeletal muscle is the primary site for glucose metabolism in the body. While it is currently unknown by which mechanism IMAT may act on metabolism, it does appear that a relationship exists between increased levels of IMAT and decreased whole body glucose metabolism particularly in those who have suffered an injury that reduces muscle function. It is theorized that the close proximity of IMAT to the muscle fiber may impair the local muscle environment through aforementioned increase in local proinflammatory cytokines [10, 59], impaired blood flow [5, 8], or increasing the rate of lipolysis within skeletal muscle resulting in an increased concentration of glucose within the skeletal muscle itself, leading to insulin resistance [5, 8].

## 4. IMAT and Muscle Function

The structural composition of muscle is an important factor in its function [23]. It is now well established that a loss of lean muscle mass in older adults does not directly translate into a loss of strength [41, 74]. The Baltimore Longitudinal Study of Aging found that while grip strength and muscle mass both declined with age, older adults were weaker than the loss of muscle mass alone would predict [74]. Similar results were found in a 3-year longitudinal study of 1800 healthy older adults. In this finding from the Health ABC Study, muscle strength declined even in those individuals who gained lean muscle mass. While lean mass decreased by approximately 1% a year, strength decreased up to 4% during the same time period [41]. This clear dissociation between lean mass and strength advocates for factors other than lean muscle mass being responsible for the declines in muscle function seen with aging. IMAT is one such factor that may impact the muscle function losses that are associated with aging.

**Figure 3:**
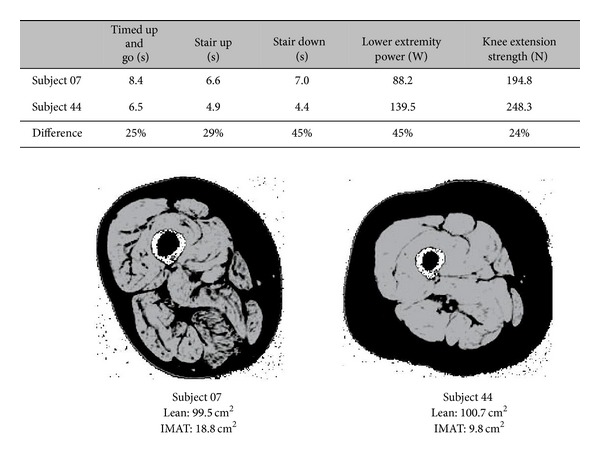
Two women with similar age, BMI, and levels of lean muscle mass but with differing levels of IMAT in a cross-sectional MRI image of the thigh. Subject 7 has double the level of IMAT (black within the muscle) in her thigh as subject 44. While both women have similar levels of lean tissue (seen in grey), they have different levels of mobility and muscle function. The increased levels of IMAT and decreased muscle and mobility function of subject 7 are consistent with literature that reports that increased levels of IMAT are associated with decreased muscle and mobility function.

An emerging body of literature supports IMAT as a significant predictor of both muscle and mobility function in older adults suggesting that increased IMAT may at least partially explain a loss of strength and mobility seen with aging [23–31] (Figure 2). Older adults with higher levels of IMAT in the legs have lower muscle strength [23, 30] as well as muscle quality [23] or the force produced per unit of cross-sectional area of muscle, as demonstrated by the two women whose thigh images are presented in Figure 3. Decreases in muscle quality may lead to difficulties in functional activities [75] and several studies have also demonstrated that adults with comorbid conditions such as COPD [45], stroke [28], osteoarthritis [76], kidney disease [77], and cognitive decline [78] demonstrate decreases in muscle quality. The relationship of increased levels of IMAT and decreased strength and muscle quality is reported in multiple studies in the thigh [23] and calf muscles [30], in healthy elders [23], and in adults with comorbid conditions including diabetes and peripheral neuropathy [30]. It is intriguing that this relationship does not appear to be confined to older adults [26]. After 30 days of single limb suspension, Manini et al. found that young (~20 years) healthy individuals experienced an increase of 15–20% in IMAT of both the calf and thigh muscles. This increase in IMAT also exceeded the loss of lean tissue suggesting that IMAT was not just merely “filling” the space left by lean tissue atrophy [26]. The increase in IMAT also accounted for a 4–6% of loss of strength, again emphasizing that IMAT is more than an inert storage depot, but may also play a role in inactivity related strength loss.

High levels of IMAT are also associated with decreased activation of the quadriceps muscles in older adults [31]. We found a moderate significant negative relationship between IMAT and quadriceps muscle activation in a small sample of older adults. Muscle activation, in this study, was quantified by the central activation ratio, a measure of a muscle's ability to fully activate during a maximal effort voluntary isometric contraction. It appears that not only may IMAT impair a muscle's ability to produce force but also it may actually hinder the improvement in muscle quality typically seen with resistance training [59]. We examined changes in muscle quality after 12 weeks (3x/week) of resistance training in 70 older adults with a history of falls and found that only individuals with low amounts of IMAT in the thigh at the start of training were able to significantly improve muscle quality. Similar to the loss of muscle quality with high levels of IMAT, a decrease in muscle activation in the presence of high amounts of IMAT suggests that IMAT may be partially responsible for inhibiting muscle force production and improvements with strength training.

## 5. IMAT and Mobility Function

Perhaps even more important than the association between IMAT and muscle function is the relationship between IMAT and mobility. There is an increasing amount of evidence linking IMAT with mobility impairment in older adults [25, 27, 29, 30, 42, 43]. Increased levels of IMAT are associated with decreased six-minute walk distance [27, 30, 79], decreased gait speed [43], decreased physical performance [25, 30], difficulty with repeated chair stands [43], and slower stair descent and timed up and go tests [27]. This relationship has consistently been reported in a variety of populations of older adults including healthy elders [43], those with a history of diabetes [25, 30], COPD [45], falls [27], and cancer [27].

IMAT is frequently associated with mobility function even when lean tissue is not suggesting that IMAT may in fact be an important variable when referring to mobility function in older adults [80]. IMAT is also predictive of future mobility limitations [42]. A large study of over 3000 older adults aged 70–79 followed up for two and one half years revealed that individuals with the greatest amounts of baseline IMAT were 50 to 80% more likely to develop mobility limitations over the following two and one half years when compared with those with the lowest levels of baseline IMAT [42]. This finding was consistent even after adjusting for baseline total body fat and muscle strength.

High levels of IMAT may not only impair mobility but also increase the risk for developing disability. Increased levels of IMAT correlate with low bone mineral density and an increased risk of hip fracture [81, 82]. IMAT levels of the mid-thigh are noted to be a strong and independent determinant of bone mineral density [82]. Additionally, the Health ABC Study, a large longitudinal investigation of over 2500 individuals between the ages of 70 and 79 years, reported a large increase in the risk for hip fracture with increased IMAT [81]. A decrease of one standard deviation of muscle density of the thigh as measured with CT conferred a 50% increase in hip fracture risk [81]. Even after adjusting for bone mineral density, an increase in IMAT raised the risk of a hip fracture by 40% [81].

It is clear that increased levels of IMAT are associated with decreased muscle and mobility function in older adults but whether IMAT is a marker of muscle dysfunction or whether it has a direct effect on muscle dysfunction is not currently known. IMAT may act as an intermediary modifying preexisting pathological process as IMAT's harmful relationship with muscle and mobility function has been theoretically attributed to an increase in proinflammatory cytokines [10, 26, 48, 65, 71] similar to the attributed effects of proinflammatory cytokines on metabolic function. Interestingly, several authors have reported relationships between increased proinflammatory cytokines and decreased muscle [83, 84] and mobility function [85–87] that are strikingly similar to those reported between IMAT and muscle and mobility function [19].

IMAT may also be harmful to muscle and mobility function due to mechanical changes in muscle that occur in the presence of IMAT that can lead to changes in muscle fiber orientation [56]. Studies of rotator cuff injuries suggest that the loss of force in a muscle may be related to increased levels of IMAT [56]. After a supraspinatus tear, elasticity of the muscle decreases and passive tension of the supraspinatus is increased. This decreased elasticity leads to a poorer ability to actively generate force, resulting in a loss of maximal tension of the muscle [88]. In addition to the loss of elasticity in rotator cuff muscles, it has been hypothesized that excess IMAT leads to an alteration in contractile fiber pennation angle, hence resulting in an unfavorable mechanical angle and a concomitant reduction in force production [56, 89]. We are unaware of studies that have examined the effect of IMAT on elasticity or of pennation angle in locomotor muscles. While the impact of IMAT relative to elasticity or pennation angle might be expected to be similar in other muscles, the results from rotator cuff studies should be interpreted cautiously due to differences in the muscle's architecture and function. Additionally, fatty infiltrate in rotator cuff muscles follows a known musculotendinous injury, that is, a rotator cuff tear. The cause of the increased fatty infiltration associated with many metabolic or systemic diseases is not as easy to pinpoint as there is no direct muscular injury. Future research should elucidate the mechanisms behind increased IMAT and decreased muscle and mobility function in older adults and importantly should determine if minimizing IMAT is accompanied by improved muscle and mobility function.

## 6. Aging, Weight Loss, Activity, and IMAT

Several authors have implied that IMAT is an unwanted but inevitable consequence of aging as epidemiological, longitudinal, and cross-sectional studies have reported significant positive relationships between aging and IMAT [7, 48, 63, 90]. Some have theorized that whole body IMAT increases as little as 9 grams/year [7] to as much as 70 grams/year [63]. The majority of studies examining the effects of aging on increases in IMAT have been small and cross-sectional and have failed to account for activity levels and disease status or have investigated only a narrow age range. These caveats call into question the definitive assertion that IMAT is an inevitable consequence of aging [7, 63, 90]. In the largest longitudinal study to date, Delmonico et al. followed up over 1600 older adults between the ages of 70 and 79 for 5-years [48]. After accounting for race, weight changes, health status, and activity levels, they found decreased thigh muscle density even in those who lost weight or were weight stable over a 5-year period. However, it should be noted that increases in IMAT were clearly influenced by increases in body weight as those who gained the most body weight over five years also gained the most IMAT. Furthermore, the study did not report the reasons for loss of body weight (i.e., illness). Weight loss due to intentional caloric restriction and exercise may have a different influence on IMAT than weight loss due to illness as numerous intervention studies have found that intentional weight loss leads to decreases in IMAT [52, 62, 91, 92].

**Figure 4:**
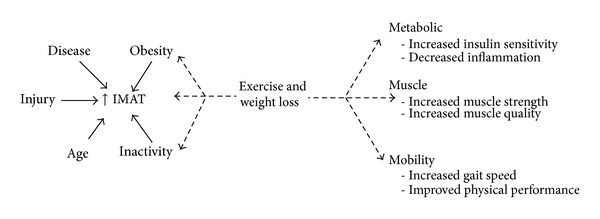
Exercise and weight loss may act to directly decrease IMAT, improve factors associated with increased IMAT such as obesity and inactivity, and improve metabolic, muscle, and mobility dysfunction.

More recent work suggests that increases in IMAT may be more a product of illness, disuse, or inactivity than aging per se [24, 29, 64]. This is a clinically important finding as it suggests that IMAT may be amenable to change via a physical activity intervention (Figure 4). Longitudinal twin studies have demonstrated that after 32 years of difference in activity habits, inactive twins had 54% higher IMAT in their mid-thigh compared to their more active twin [35]. High levels of spasticity after spinal cord injury have also been shown to protect against the accumulation of IMAT [57]. Further support for the assertion that physical activity has a strong influence on IMAT is found in studies of young, healthy adults following periods of inactivity [26], when comparing younger to older athletes [14, 64] and when comparing obese active to inactive individuals [29]. After 30 days of single limb suspension, a method of immobilizing one leg, young, healthy adults demonstrate an increase of 15% IMAT in the immobilized thigh and 20% in the calf [26]. In a cross-sectional study examining master athletes from age 40 to 81 who consistently participated in high levels of physical activity it was found that younger and older adults did not differ in IMAT levels [26]. Even in a population of obese adults with diabetes and peripheral neuropathy, conditions known to be associated with increased IMAT, there still exists a significant relationship between the number of steps taken in a day and the volume of IMAT in the calf [29]. Tuttle et al. reported that the average daily step count was able to explain up to 19% of the variance in IMAT in the calf of older adults with diabetes and peripheral neuropathy [29]. Based on these studies, it appears that IMAT may be amenable to change via increasing physical activity levels. However, the magnitude of changes reported questions the clinical significance of these changes. It may be that significant weight loss, via physical activity or diet, may be necessary to achieve meaningful changes in IMAT.

Multiple studies have examined the effects of diet, exercise, or a combination of diet and exercise on IMAT [20, 22, 24, 51, 52, 55, 59, 61, 62, 91–100]. Most have reported decreased IMAT following intervention [20, 22, 51, 52, 55, 61, 62, 91–94, 96, 97, 99]. The current general consensus among studies examining changes in IMAT with weight loss alone or with exercise is that weight loss is necessary to see significant changes in IMAT [20, 51, 52, 55, 62, 91–93, 97]. However, it is possible that exercise, when performed at a sufficient intensity and duration to induce weight loss, is actually superior at decreasing IMAT levels compared to weight loss induced by reduced calorie intake [55, 62, 97]. Murphy et al. compared the effects of exercise induced weight loss to weight loss induced by calorie restriction alone in overweight adults aged 50–60 [62]. They found that when exercise resulted in weight loss, the loss of IMAT was two times greater than calorie restriction alone. This finding is in agreement with Christiansen et al. who found that the combination of calorie restriction and exercise resulted in an 11% decrease in IMAT while calorie restriction alone resulted in a 7% decrease in IMAT [55]. While weight loss may be necessary to decrease IMAT, this may not be a desirable option for some older adults. Weight loss in frail, older adults with already low body mass indexes may be accompanied by loss of muscle mass and function and therefore may not result in a positive outcome. There is currently a paucity of literature that examines the effects of any intervention on IMAT in frail, older adults. Most studies of IMAT to date have examined younger [22, 55, 92, 95, 96], obese, [22, 52, 55, 91–93], or overweight [20, 22, 51, 52, 62, 91, 94–96, 101] populations, making generalization to frail, older adults difficult. Goodpaster et al. reported that physical activity nearly ameliorated the increase in IMAT that occurs with sedentary behavior in older adults with a mean age of 76 years [24]. A modest walking program of 1-2 times per week for as little as 30 minutes per session stabilized IMAT accumulation in these individuals. In contrast, in this same study, the control group that did not participate in any formal exercise program experienced an 18% gain in thigh IMAT over 12 months [24]. This suggests that physical activity might mitigate the accumulation of IMAT in older adults. However, only two small studies have found that increasing physical activity, one through walking and the other through resistance training, decreased IMAT in this population [93, 94]. We also found that resistance training decreased IMAT in the thigh muscles of older adults (~65 years) who had a CVA [101]. This is a promising finding as it suggests that IMAT may respond to physical activity interventions, even in older adults with comorbid health conditions. However, more research is needed to (1) verify these findings, (2) determine the most effective method of reducing IMAT, and (3) assess the clinical impact of doing so in older adults.

## 7. Future Directions and Rehabilitation Considerations

More work is necessary to determine the role of increased IMAT on metabolic, mobility, and muscle dysfunction. It has not yet been determined if IMAT is merely a marker of dysfunction or if it has some direct or indirect role in modifying metabolic, muscle, and mobility function. If IMAT does impair muscle activation, then using exercise as a method to reduce IMAT may have a limited effect particularly in frail, older adults. Impaired muscle contraction may minimize the muscles ability to mobilize and utilize IMAT as a fuel source, and it is possible that a combination of therapies will be necessary to reduce IMAT. This may be particularly true in frail, older adults with limited ability or need to change their body mass. The addition of electrical stimulation to exercise may be one method to reduce IMAT and improve muscle function. In a small study of nine individuals with complete spinal cord injury which compared the use of electrical stimulation on the quadriceps muscles twice a week for 12 weeks combined with calorie restriction to calorie restriction alone, the addition of electrical stimulation was shown to significantly decrease IMAT [58]. While the decrease in IMAT was still relatively small (approximately 3%); particularly noteworthy is the observation that the calorie restriction group increased IMAT by 3% during this same period of time [58]. The use of electrical stimulation may result in increased muscle contraction and perhaps an increased ability to use IMAT as a fuel source thus decreasing IMAT within the muscle. This has yet to be explored and is currently only speculative.

Another promising direction that may yield new therapeutic targets is research into the origins of IMAT. Studies investigating the cellular origins of IMAT [102, 103] are attempting to determine the cellular processes that precipitate increased IMAT. While these origins are currently unknown, if found to be similar to other ectopic fat depots such as those found in the liver, pharmacological interventions used in combination with exercise may be a treatment option worth future exploration [72]. Current recommendations for the treatment of nonalcoholic fatty liver disease that results in the accumulation of fat within the liver, similar to IMAT accumulation in the muscle, include the combination of diet, exercise, and in some cases medication [72]. While we are unaware of any trials examining the effects of medication on IMAT, the use of anti-inflammatory or other medications that have been effective at treating other ectopic fat depots such as thiazolidinediones may be useful in the treatment of IMAT, particularly in older frail adults [72].

Large randomized control trials examining the effect of exercise on decreasing IMAT are limited, though it does appear that physical activity, at a minimum, may serve as a preventive strategy to halt the infiltration of IMAT into muscle [24] and may even decrease IMAT in muscles that have already undergone this abnormal adaptation [20, 22, 51, 52, 55, 61, 62, 91–94, 96, 97, 99]. The majority of studies that have demonstrated a decrease in IMAT have been studies that employed a combination of calorie restriction and aerobic exercise for at least 6 months [20, 51, 52, 62, 91, 93, 96]. It also appears that resistive exercise alone [94, 101] or in combination with weight loss [97] or aerobic exercise [55, 61] may decrease IMAT.

It is theorized that exercise training may access IMAT as a fuel source during times of increased activity of the muscle [20, 30]. While speculative, IMAT may be preferentially metabolized as a fuel source to support the increased demands of the muscle thus resulting in a decrease of IMAT with long-term activity [20, 30]. While exercise should be a lifelong activity, to decrease IMAT levels a minimum of 12 weeks of intervention appears to be required to decrease IMAT, though 6 months may be superior. It is important to note that exercise interventions have multiple effects on physiology and the improvements found in these studies may not be due to a reduction in IMAT. Further research is needed to elucidate the role of decreased IMAT on muscle and metabolic function as well as the most effective exercise prescription to target a reduction in IMAT in older adults.

As our population ages and larger number of individuals with metabolic, muscle, and mobility dysfunction require effective interventions, there is an increase in the need for understanding and treating the multiple negative metabolic and muscle adaptations that may occur. IMAT is now recognized as an important predictor of muscle metabolism and function and also appears to be a modifiable muscle risk factor. Exercise and physical activity appear to be effective countermeasures against increases in IMAT. Future research should focus not only on the causes and mechanisms of increased fatty infiltration but also on establishing whether and how IMAT is involved in the development of the pathologies discussed as well as effective intervention regimes to decrease IMAT.
